# The Relative Age Effect on Competition Performance of Spanish International Handball Players: A Longitudinal Study

**DOI:** 10.3389/fpsyg.2021.673434

**Published:** 2021-06-29

**Authors:** Alfonso de la Rubia, Alberto Lorenzo, Christian Thue Bjørndal, Adam Leigh Kelly, Abraham García-Aliaga, Jorge Lorenzo-Calvo

**Affiliations:** ^1^Departamento de Deportes, Facultad de Ciencias de la Actividad Física y del Deporte—Instituto Nacional de Educación Física, Universidad Politécnica de Madrid, Madrid, Spain; ^2^Department of Sport and Social Science, Norwegian School of Sport Sciences, Oslo, Norway; ^3^Department of Sport and Exercise, Research Centre for Life and Sport Sciences, Birmingham City University, Birmingham, United Kingdom

**Keywords:** relative age effect, birthday effect, team sport, competition performance, talent identification, talent development, elite sport policy

## Abstract

**Background:** Competitive success is the ultimate objective of elite professional sport organisations. Relative age effects (RAE) impact athlete selection processes in the short and long-term performance. The aims of this study were: (i) examine the presence of RAE by gender, competitive level, and playing position, as well as evaluate the impact of RAE on individual (goals, percentage of effectiveness in shots, saves; percentage of effectiveness in saves, assists, turnovers, steals, blocked shots, penalties, minutes played, and minutes played per match) and collective competition performance (final team position); and (ii) analyse the impact of RAE on the evolutionary trends of individual performance in international competitions throughout 16 seasons in Spanish handball (2005–2020).

**Methods:** The sample included 631 Spanish handball players (male: *n* = 359; female: *n* = 272). A Chi-square goodness-of-fit test was used to assess whether a skewed birthdate distribution occurred. A one-way analysis of variance (ANOVA) of independent measures was used to examine the individual and collective statistical parameters by birth quartiles. A linear regression in a Hopkins sheet were performed to compare individual performance trends.

**Results:** The results revealed RAE in the male formative categories (*p* < 0.001), as well as the male and female senior categories (*p* < 0.05). By position, RAE especially affected the “centre-back” in the male formative (*p* < 0.01) and senior categories (*p* < 0.05). No significant relationship between RAE and individual performance was found in male formative categories, while an impact of RAE on the “minutes played” was detected in the female senior category (*p* < 0.05). With regard to collective performance, a higher number of relatively older handball players was observed in the best ranked teams in the male formative categories and in the quarter-final teams in the female formative categories (*p* < 0.05). Among the male players, relatively older players spent more minutes on the court than relatively younger players, although this advantage dissipated over time and did not lead to better performance. Among the female players, relatively younger players were found to perform better as the level of competitive handball increased.

**Discussion:** These findings are important for talent identification and development policies in sport federations and other elite sport institutions by demonstrating the many unintended consequences of selections to international competitions at the youth level.

## Introduction

Developmental pathways are mapped by national sport federations to prepare young athletes for the demands of adult international competition (De Bosscher et al., 2006). In Spanish handball, the talent identification (TID) processes in national youth teams begin when female players are aged 16 years and male players are aged 17 years (although scouting and try-outs may occur earlier) (Bjørndal et al., 2018a). These youth development stages are organised into competition cycles for the duration of 2 years using a pre-established cut-off dates of January 1. Based on the timing of an athlete's birthdate within a given cohort, an individual can be relatively older or younger in comparison to his or her peers (Musch and Grondin, 2001). The biological and maturational differences among athletes born almost 12 months apart are actualized in physical, anthropometric, and physiological advantages, and could thus lead to a development disadvantage for some athletes; especially those born at the end of the selection year (Vaeyens et al., 2005). This is known as “relative age effects” (RAE), which can impact upon immediate, short-term, and long-term inequalities in the participation, selection, and attainment in handball (Barnsley et al., 1985).

RAE have been well-documented in team sports (Wattie et al., 2008), whereby an overrepresentation of relatively older athletes (e.g., those born in the first months of the selection year) compared to those who are relatively younger (e.g., those born in the latter months of the selection year) is a common theme in youth sport (Cobley et al., 2009). This phenomenon is particularly evident at higher levels of competition when athletes are selected into talent pathways (Baker et al., 2009). However, the impact of RAE may not necessarily be consistently strong throughout an athlete's development, whereby it often decreases as the chronological age of an athlete increases, and RAE may plateau as they reach adulthood (Brustio et al., 2018).

Variations in the impact of RAE on an athlete's career may also be affected by factors such as: (a) playing position (Fonseca et al., 2019; Pino-Ortega et al., 2020)—relatively older players tend to be overrepresented in the playing positions with higher physical and conditional demands (López-del-Río et al., 2019) or where certain key psychological characteristics such as leadership or self-confidence are needed (Chittle et al., 2017); (b) the role of coaches (Krahenbühl and Leonardo, 2020)—the positive perception of a player's performance in the formative stages can be decisive in order to be able to enjoy favourable competitive conditions and, thus, have a better chance of achieving high sport performance; (c) and/or individual characteristics (Camacho-Cardenosa et al., 2018)—the combined influence of the RAE with other variables, such as height or handedness, results, among other examples, in an overrepresentation of relatively older and taller basketball players or a high proportion of left-handed handball players born in the first months of the year at elite levels (Schorer et al., 2009; Rubajczyk et al., 2017).

RAE impact not only player selection, but the short-term and long-term competition performance of athletes (de la Rubia et al., 2020b; Kalén et al., 2020). As an example, short-term statistical parameters suggest that RAE can impact on individual performance, especially among young male players (Arrieta et al., 2016; Ibañez et al., 2018; de la Rubia et al., 2020a). Greater maturation development (i.e., see the “maturation-selection hypothesis” by Baker et al., 2010) in conjunction with unequal recruitment based on physical and anthropometric criteria, appears to lead to better sports performance among relatively older players (Jackson and Comber, 2020). However, in the context of international competitions for males, the impact seem less clear. International competitions are specialised high-performance sport contexts, and it might naturally be assumed that player selections would be influenced primarily by technical-tactical criteria (Karcher et al., 2014), a player's initial selection age, or the country's long-term performance (Kalén et al., 2020). The lack of impact of RAE on performance in female competitions has been explained by lower biological and conditional differences among players from the same selection year (Konstantinos et al., 2018) and by the “depth of competition hypothesis” (Baker et al., 2009). With regard to long-term competition performance, the trend seems to be varied or reversed (Kelly et al., 2021). Thus, relatively young players could achieve high performance, to a greater extent, if they manage to overcome the initial difficulties (McCarthy et al., 2016). This could be due, among other factors, to a higher development of specific technical-tactical skills (Güllich and Emrich, 2014), a lower injury rate (Bjørndal et al., 2018a), and relatively younger players having to grow from the adverse experiences of being initially disadvantaged (Collins and MacNamara, 2012).

RAE have been investigated using cross-sectional studies based on official statistics from specific competitions, such as the Handball World Championships. Individually, at the World Handball Championships held between 2013 and 2017, for example, de la Rubia et al. (2020a) showed that relatively older players (with the exception of the female senior category) played for more minutes than their relatively younger peers; although this does not necessarily results in better performance. However, Karcher et al. (2014) found that RAE had a limited impact on playing time, which varied primarily by playing position. Collectively, Saavedra and Saavedra (2020) identified an association between RAE and the final position of the female teams in the top eight places in the 2018 Women's Youth World Championship. A higher number of female players in these teams were also born in the first selection year compared to teams in lower positions. In contrast, however, Fonseca et al. (2019) did not detect any relationship between RAE and the final position of the teams in the male Youth World Handball Championship held in 2017. Nevertheless, there has also been a proliferation of longitudinal studies focusing on talent identification and development (TID) programmes designed by national sport federations. International competitions have been found to have a predominance of relatively older players. For instance, an overrepresentation of relatively older players on team season rosters was found in the German Handball Federation (DHB) between 1993 and 2007 (Schorer et al., 2013), in the Danish Handball Federation (DHF) between 2003 and 2017 (Wrang et al., 2018), and the Norwegian Handball Federation (NFH) between 2004 and 2017 (Bjørndal et al., 2018a).

RAE in team sports has been analysed using cross-sectional studies, cross-sectional studies of particular different ages in the same sport, and longitudinal studies (Dixon et al., 2020). To our knowledge, RAE in handball has not been evaluated using a combination of these three approaches together, using statistical performance parameters. Therefore, the broad aim of this study was to analyse the influence of RAE on the participation and performance of Spanish male and female handball players in official international competitions throughout 16 seasons (2005–2020). The specific aims were to use different methodological approaches to: (i) examine the prevalence of RAE at different competitive levels (formative and senior categories) and playing positions (i.e., goalkeeper, wing, centre-back, back, and pivot), and to evaluate the impact of RAE on individual and collective competition performances using official statistical parameters (a cross-sectional approach); and, (ii) analyse the long-term impacts of RAE using individual statistical parameters during the period 2005–2020 to observe possible technical-tactical performance trends (a longitudinal approach).

## Materials and Methods

### Participants

The sample consisted of 631 handball players (male: *n* = 359; female: *n* = 272). All the players were selected by the Royal Spanish Handball Federation (RFEBM) to be part of the Spanish national teams which aimed to compete in the World Handball Championship organised by the International Handball Federation (IHF) during the 2005–2006 and 2019–2020 seasons. The competition categories we analysed corresponded to the official categories defined by the IHF for international tournaments, including: Female U-18 (*n* = 63), U-20 (*n* = 96), and senior (*n* = 113), as well as Male U-19 (*n* = 109), U-21 (*n* = 119), and senior (*n* = 131). For the subsequent analysis, the players were grouped in “formative categories” (U-18/U-19 and U-20/U-21) or the “senior category.”

According to the biannual competition cycles established by the IHF (January 1 as a cut-off date), players categorised as “minors” (those who were selected despite being younger than the category selection year) were not included in the study sample in order not to duplicate data on date of birth in different variables (Steingröver et al., 2017). Thus, 3.8% of male players (U-19, *n* = 4; U-21, *n* = 10) and 0.4% of female players (U-18, *n* = 1) were excluded. Players in the formative categories (U-18/U-19 or U-20/U-21) could also be included later in the senior categories. Moreover, players were categorised by the playing position (goalkeeper: *n* = 85; wing: *n* = 160; back: *n* = 183; centre-back: *n* = 98; pivot: *n* = 105). Additionally, the birthdate distribution of the wider Spanish population between the ages of 15 and 40 years (i.e., the minimum and maximum age of the selected Spanish players) were extracted from the website of the National Institute of Statistics (INE) (https://www.ine.es/jaxi/Tabla.htm?path=/t20/e245/p08/l0/&file=01002.px) for comparison.

### Procedures

Official player data were extracted from the “Competitions Archive” section of the IHF's official website (https://archive.ihf.info/en-us/ihfcompetitions/competitionsarchive.aspx) and verified by consulting the online library of the Royal Spanish Handball Federation (RFEBM) (https://www.rfebm.com/biblioteca). All the players' names were anonymised before analysis. No informed consent or ethical approval was required because the data were publicly accessible. The inclusion criteria for the selected seasons were to: (1) provide a reasonably wide sample of handball players (*n* = 631) from which to draw generalisable conclusions; (2) present a comprehensive database that could provide information about both handball players' profiles and competition performances (i.e., both individual and collective statistics); (3) analyse the only competition periods in which the World Handball Championships had taken place throughout the same biannual competition cycle in the three official competition categories (the Youth World Handball Championship competition was established in 2005 for males and in 2006 for females). We decided not include the European Youth Olympic Festival, the Youth Olympic Games, and the Olympic Games in the analysis because they did not meet any of these criteria.

Players were categorised in quartiles (Q), based on their birthdate and according to the competition cycles used by the IHF (“Fixtures and Results/Team Roster” section of the IHF website). In the formative categories, youth (U-18/U-19) and junior (U-20/U-21) players born in the first selection year (even years) of the biannual competition cycle were grouped as follows: between January 1 and March 31–Quartile 1 (Q1); between April 1 and June 30–Quartile 2 (Q2); between July 1 and September 30–Quartile 3 (Q3); between October 1 and December 31–Quartile 4 (Q4). Likewise, youth (U-18/U-19), and junior (U-20/U-21) players born in the second selection year (odd-numbered years) of the biannual competition cycle were grouped as follows: between January 1 and March 31–Quartile 5 (Q5); between April 1 and June 30–Quartile 6 (Q6); between July 1 and September 30–Quartile 7 (Q7); between October 1 and December 31–Quartile 8 (Q8). Only quartiles Q1–Q4 were applied when classifying senior players according to annual competition cycle. Additionally, the players were classified according to the playing position: “goalkeeper” (GK), “wing” (W), “back” (B), “centre-back” (CB), and “pivot” (P).

Individual and collective competition performance data were extracted from the “Fixtures and Results/Cumulative Statistics” section of the RFEBM website. The reliability of the data recording was checked by a second external observer handball coach with 5 years of experience. Thus, the observer was trained by means of an observation manual on the concordance agreement (Anguera, 1990) based on the kappa coefficient (Cohen, 1960). According to the kappa coefficient rating scale (Landis and Koch, 1977), all the recording variables presented indices equal to or >0.84 (almost perfect agreement), demonstrating the validity and reliability of the data recording and analysis process.: The statistical parameters and the Kappa coefficient associated with individual and collective competition performance were: (a) “goals” (Go; κ = 0.95); (b) “the percentage of effectiveness in shot” (PeSh; κ = 0.92); (c) “saves” (Sa; κ = 0.90); (d) “the percentage of effectiveness in saves” (PeSa; κ = 0.88); (e) “assists” (As; κ = 0.87); (f) “turnovers” (To; κ = 0.86); (g) “steals” (St; κ = 0.94); (h) “blocked shots” (BS; κ = 0.91); (i) “penalties” (Pen; κ = 0.84); (j) “time played” (Min; κ = 0.90); (k) “time played per match” (Min-M; κ = 0.90), and (l) “championship ranking” (Cl; κ = 1.00). The number of handball penalties was quantified as the sum of the number of yellow cards (x1) plus the number of exclusions (x2) plus the number of red/blue cards (x3). Collective performance was determined by the final team position at the end of each respective World Handball Championship.

### Statistical Analysis

Data analysis was conducted using the Statistical Package for Social Sciences (SPSS 23.0, IBM Corp., Armonk, NY, USA). The differences between the observed and expected birthdate distributions (independent category) according to different dependent variables (gender, competition category, playing position and final team position) were tested using the chi-square goodness of fit test (χ^2^), providing data with regard to absolute frequency (*n*), relative frequency (%) and degrees of freedom (*df*). By checking the homogeneous sample distribution of the Spanish population, the expected frequency of births in a quartile of 25% was established. Odds ratios (*OR*) were calculated for the quartile distributions to examine subgroup differences with respect to the potential non-uniformity of the birthdate distribution. The odds ratios compared the birthdate distribution of a particular quartile (Q1, Q2, Q3, Q4, Q5, Q6, or Q7) with the reference group of the relatively younger players (Q4/Q8). In order to determine the strength of association, a Cramer's V (*Vc*) was applied, in which 0.10–0.20 indicated a “weak association”; 0.20–0.40, a “moderate association”; 0.40–0.60, a “relatively strong association”; 0.60–0.80, a “strong association”; and, *Vc* > 0.80, a “very strong association” (Cohen, 1998). The level of significance was *p* < 0.05.

A one-way analysis of variance (*ANOVA*) of independent measures was used to examine the effects of RAE on the individual statistical parameters according to gender and competitive level. A Tukey *post-hoc* test was used to assess the differences within each group. Normality and homogeneity were verified through the Kolmogorov-Smirnov test (*K-S*) and the Levene test (homogeneous variances). Data were presented as the mean ± standard error (*X* ± *SD*), statistical value (*F*), and effect size (η^2^). The level of statistical significance was *p* < 0.05.

A linear regression was conducted to verify the impact of RAE (independent variable) on the individual performance (dependent variables) throughout the analysed period (2005–2020). Moreover, a Hopkins monitoring and reliability spreadsheet (Hopkins, 2017) was used to observe the qualitative inference of the independent variable (birthdate), expressing the data through the mean ± standard deviation (*X* ± *SD*) and the slope of the linear regression (*y* = *m x* + *n*). Positive values of the slope mean an upward trend of the analysed statistical parameter, while negative values indicate a downward trend. The real possibilities of change in the performance variables were classified as follows: “<1%” = very unlikely increase/decrease; “1–5%” = unlikely trivial increase/decrease; “5–25%” = trivial increase/decrease “25–75%” = increase/decrease; “75–95%” = substantial increase/decrease; “95–99%” = likely substantial increase/decrease; and, “>99%” = very likely increase/decrease. The Hopkins' spreadsheet has been used in other studies of team sports to analyse variations in competition performance (Ward et al., 2018; Lorenzo et al., 2019) or physical performance (Pliauga et al., 2018; Ruf et al., 2018; Ferioli et al., 2020) over a period of time.

## Results

### RAE—Gender, Competitive Level, and Playing Position

The quartile distribution of the birthdates, by competitive level, in each of the nine biannual cycles in the 2005–2020 seasons is shown in Table 1. A higher number of players born in the even-numbered years of the biannual competition cycle was observed in the formative categories (*n* = 249; 64.34%). The quartiles with the most representation of players in the formative categories were in the U-18/U-19 and U-20/U-21 categories: Q1 *n* = 71 (18.35%) and Q2 *n* = 64 (16.54%). In the senior category, the quartiles with the most representation of players were: Q1 *n* = 80 (32.79%) and Q4 *n* = 63 (25.82%).

**Table 1:** Quarterly distribution (“*n*” and “%”) of birthdates by competition level and biannual competition cycle.

**BCC**	**Q1**	**Q2**	**Q3**	**Q4**	**Q5**	**Q6**	**Q7**	**Q8**
	***n(%)***	***n(%)***	***n(%)***	***n(%)***	***n(%)***	***n(%)***	***n(%)***	***n(%)***
	**Formative categories (U-18/U-19 and U-20/U-21)**							
2005-06	4(13.3)	4(13.3)	5(16.7)	3(10.0)	7(23.3)	2(6.7)	4(13.3)	1(3.3)
2007-08	11(18.0)	13(21.3)	11(18.0)	10(16.4)	2(3.3)	6(9.8)	3(4.9)	5(8.2)
2009-10	18(29.5)	8(13.1)	7(11.5)	9(14.8)	3(4.9)	4(6.6)	3(4.9)	9(14.8)
2011-12	9(19.1)	3(6.4)	7(14.9)	10(21.3)	4(8.5)	7(14.9)	4(8.5)	3(6.4)
2013-14	8(26.7)	4(13.3)	5(16.7)	3(10.0)	4(13.3)	3(10.0)	2(6.7)	1(3.3)
2015-16	8(12.9)	12(19.4)	7(11.3)	6(9.7)	11(17.7)	6(9.7)	7(11.3)	5(8.1)
2017-18	8(12.7)	12(19.0)	11(17.5)	9(14.3)	6(9.5)	6(9.5)	6(9.5)	5(7.9)
2019-20	5(15.2)	8(24.2)	6(18.2)	5(15.2)	4(12.1)	0(0)	1(3.0)	4(12.1)
	**Senior category**							
2005-06	5(31.3)	5(31.3)	3(18.8)	3(18.8)	-	-	-	-
2007-08	13(40.6)	9(28.1)	6(18.8)	4(12.5)	-	-	-	-
2009-10	10(31.3)	9(28.1)	5(15.6)	8(25.0)	-	-	-	-
2011-12	9(28.1)	8(25.0)	6(18.8)	9(28.1)	-	-	-	-
2013-14	10(30.3)	9(27.3)	4(12.1)	10(30.3)	-	-	-	-
2015-16	12(37.5)	8(25.0)	3(9.4)	9(28.1)	-	-	-	-
2017-18	12(36.4)	4(12.1)	8(24.2)	9(27.3)	-	-	-	-
2019-20	9(26.5)	5(14.7)	9(26.5)	11(32.4)	-	-	-	-

“*BCC,” biannual competition cycle; “Q1-Q4/Q8,” birth quarter*.

**Figure 1:**
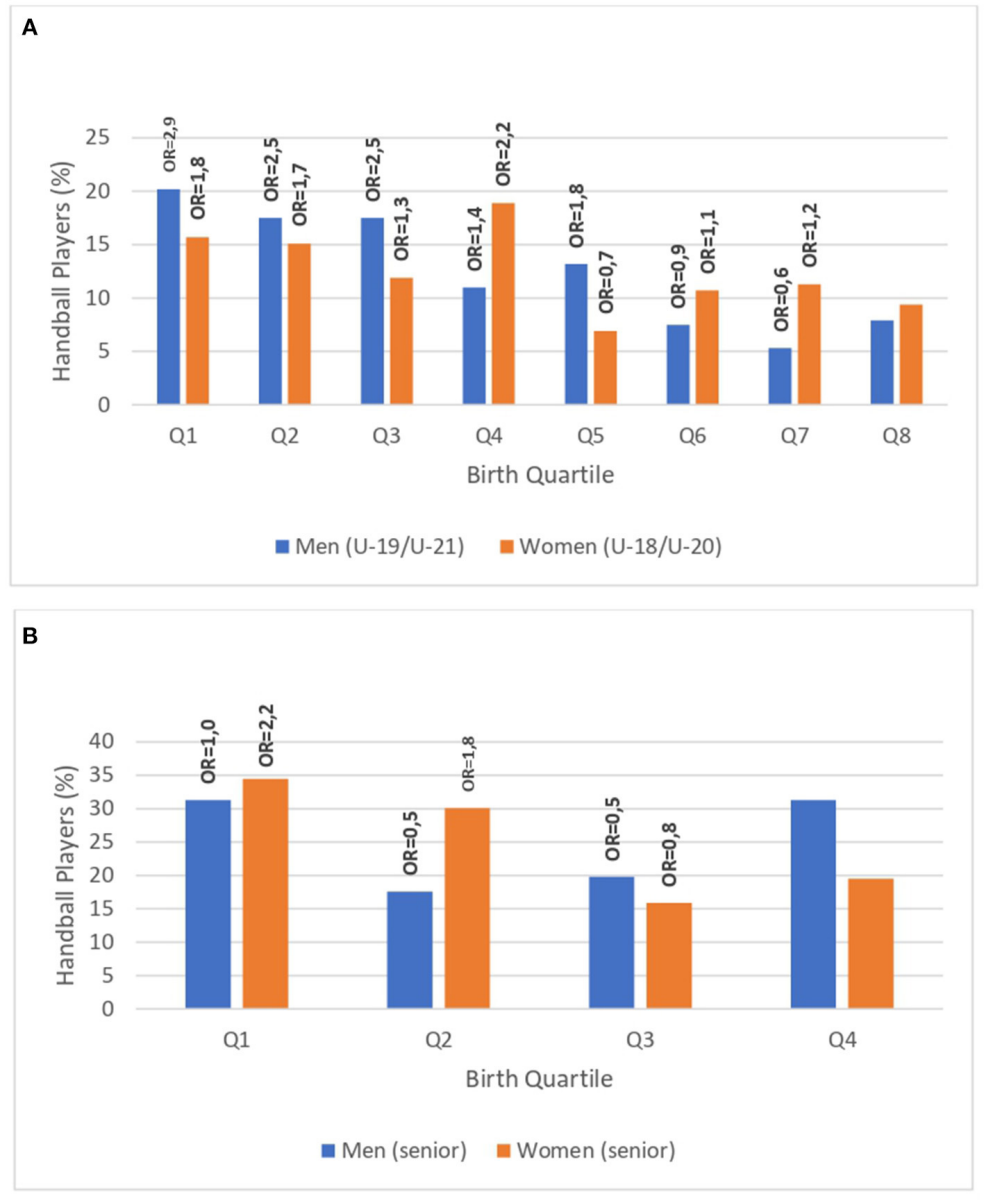
**(A)** Quarterly distribution (% and OR) of birth dates by gender in formative categories. **(B)** Quarterly distribution (% and OR) of birth dates by gender in senior category.

Figures 1A,B present the quartile distribution and “*OR*” (Q1 to Q8/Q4) of the Spanish handball players by gender in the formative categories and the senior category of the national team throughout 16 seasons, respectively. RAE was evident in male formative categories [χ^2^*(7)* = 38.60, *p* < 0.001], as well as the male [χ^2^*(3)* = 8.45, *p* < 0.05] and female [χ^2^*(3)* = 10.36, *p* < 0.05] senior categories. The largest effect sizes were identified in the male formative categories, whilst a moderate association was evident between variables (*Vc* = 0.41). RAE were not found in the female formative categories (*p* > 0.05).

In the analysis of RAE on playing positions (Table 2), an overrepresentation of relatively older players (born in Q1) was found in the following cases: “goalkeeper” in male formative categories [χ^2^*(7)* = 16.93, *p* < 0.05]; “wing” in male formative [χ^2^*(7)* = 19.19, *p* < 0.01] and female senior categories [χ^2^*(3)* = 11.25, *p* < 0.05]; “centre-back” in male formative [χ^2^*(7)* = 18.67, *p* < 0.01] and senior categories [χ^2^*(3)* = 9.67, *p* < 0.05]. An overrepresentation of relatively younger players (which runs contrary to the expected RAE) was detected among players in the “back” position in the male senior category [χ^2^*(3)* = 17.06, *p* < 0.01]. The greatest effect size was found in the “goalkeeper” position in the male formative categories (*Vc* = 0.75), and a strong association was identified. Equally strong effect sizes were found in the “centre-back” position in the male formative categories (*Vc* = 0.72), as well as the “centre-back” (*Vc* = 0.63) and “back” (*Vc* = 0.72) positions in the male senior category. RAE were not detected in any playing position in the female U-18/U-20 formative categories (*p* > 0.05).

**Table 2:** Quarterly distribution (*n* and %) for playing position by gender and competitive level.

**GEN**	**POS**	**Q1**		**Q2**		**Q3**		**Q4**		**Q5**		**Q6**		**Q7**		**Q8**		**χ^2^**	***df***	***p***	***Vc***
		***n***	***%***	***n***	***%***	***n***	***%***	***n***	***%***	***n***	***%***	***n***	***%***	***n***	***%***	***n***	***%***				
M		**Formative categories (U-19/U-21)**																			
	GK	4	13.3	3	10.0	11	36.7	2	6.7	3	10.0	2	6.7	2	6.7	3	10.0	16.93	7	0.018	0.75
	W	6	11.1	15	27.8	8	14.8	6	11.1	10	18.5	5	9.3	2	3.7	2	3.7	19.19	7	0.008	0.60
	L	17	24.3	7	10.0	9	12.9	11	15.7	8	11.4	7	10.0	5	7.1	6	8.6	11.60	7	0.115	0.41
	CB	9	25.0	6	16.7	6	16.7	3	8.3	9	25.0	1	2.8	1	2.8	1	2.8	18.67	7	0.009	0.72
	P	10	26.3	9	23.7	6	15.8	3	7.9	0	0	2	5.3	2	5.3	6	15.8	11.74	6	0.068	0.56
		**Senior category**																			
	GK	5	27.8	10	55.6	0	0	3	16.7	-	-	-	-	-	-	-	-	4.33	2	0.115	0.49
	W	11	34.4	6	18.8	8	25.0	7	21.9	-	-	-	-	-	-	-	-	1.75	3	0.626	0.23
	L	8	24.2	3	9.1	4	12.1	18	54.5	-	-	-	-	-	-	-	-	17.06	3	0.001	0.72
	CB	11	45.8	4	16.7	8	33.3	1	4.2	-	-	-	-	-	-	-	-	9.67	3	0.022	0.63
	P	6	25.0	0	0	6	25.0	12	50.0	-	-	-	-	-	-	-	-	3.00	2	0.223	0.35
W		**Formative categories (U-18/U-20)**																			
	GK	9	40.9	0	0	2	9.1	4	18.2	3	13.6	1	4.5	0	0	3	13.6	10.73	5	0.057	0.70
	W	5	11.9	9	21.4	9	21.4	7	16.7	2	4.8	3	7.1	5	11.9	2	4.8	10.95	7	0.141	0.51
	L	4	8.2	9	18.4	5	10.2	9	18.4	3	6.1	9	18.4	4	8.2	6	12.2	7.33	7	0.396	0.39
	CB	5	20.8	2	8.3	3	12.5	6	25.0	2	8.3	2	8.3	3	12.5	1	4.2	6.67	7	0.464	0.53
	P	2	9.1	4	18.2	0	0	4	18.2	1	04.5	2	9.1	6	27.3	3	13.6	5.36	6	0.498	0.49
		**Senior category**																			
	GK	7	46.7	1	6.7	7	46.7	0	0	-	-	-	-	-	-	-	-	4.80	2	0.091	0.57
	W	11	34.4	14	43.8	2	6.3	5	15.6	-	-	-	-	-	-	-	-	11.25	3	0.010	0.59
	L	9	29.0	11	35.5	5	16.1	6	19.4	-	-	-	-	-	-	-	-	2.94	3	0.402	0.31
	CB	6	42.9	3	21.4	2	14.3	3	21.4	-	-	-	-	-	-	-	-	2.57	3	0.463	0.43
	P	6	28.6	5	23.8	2	9.5	8	38.1	-	-	-	-	-	-	-	-	3.57	3	0.312	0.41

“*GEN,” gender; “M,” men; “W,” women; “POS,” playing position; “GK,” goalkeeper; “W,” wing; “LB,” back; “CB,” centre-back; “P,” pivot; “Q1-Q4/Q8,” birth quarter; “χ^2^,” Chi square; “df,” degrees of freedom; “p,” level of significance; “Vc,” Cramer's V*.

### RAE—Impact on Individual and Collective Performance

Table 3 shows the impact of RAE on individual performance by gender and competitive level throughout the period analysed (2005-2020). An impact of RAE on the “percentage of effectiveness in shots” (*p* < 0.05) was identified in the male formative categories, as well as on “turnovers” (*p* < 0.01), “steals” (*p* < 0.01), and “penalties” (*p* < 0.01) in the male senior category. In competitions for females, RAE affected the number of “steals” (*p* < 0.01), “penalties” (*p* < 0.01), “time played” (*p* < 0.05), and the “time played per match” (*p* < 0.01) in the senior category. *Post-hoc* analysis revealed that male U-19/U-21 players born in Q2 scored a higher “percentage of effectiveness in shots” than those born in Q3, whereas relatively younger players in the male senior category performed better in “steals.” However, players born in Q4 registered more “turnovers” and “penalties.” In the female senior category, relatively older players (born in Q1 and Q2) played a greater number of total minutes (“min”) and per match (“min-m”) than relatively younger players. In the U-18/U-20 female competitions, the impact of RAE on individual statistics was not detected (*p* > 0.05).

**Table 3:** Impact of the birth quartile (Q) on individual performance statistics (X ± SD) in Spanish handball players.

	**Men**									
	**Formative Categories (U-19/U-21)**					**Senior Category**				
**Statistics**	**X ± SD**	**Q**			**Differences**	**X ± SD**	**Q**			**Differences**
		**F**	***p***	*****η**^**2**^***			**F**	***p***	*****η**^**2**^***	
Go	14.69 ± 12.07	0.57	0.777	0.020	n.s.	17.54 ± 13.20	1.54	0.209	0.038	n.s.
PeSh	53.59 ± 24.87	2.40	<0.05	0.077	Q2 > Q3	54.92 ± 25.83	1.95	0.125	0.047	n.s.
Sa	47.90 ± 28.19	1.22	0.349	0.364	n.s.	52.89 ± 22.35	0.14	0.868	0.022	n.s.
PeSa	32.65 ± 6.59	0.72	0.660	0.251	n.s.	34.44 ± 3.42	1.57	0.245	0.195	n.s.
As	4.24 ± 5.15	0.65	0.712	0.022	n.s.	9.19 ± 9.32	2.23	0.089	0.054	n.s.
To	5.87 ± 5.25	1.35	0.227	0.045	n.s.	6.34 ± 5.59	4.11	<0.01	0.095	Q4 > Q2
St	0.57 ± 1.38	1.21	0.298	0.040	n.s.	3.60 ± 3.50	5.30	<0.01	0.119	Q4 > Q2; Q4 > Q3
BS	0.30 ± 1.75	0.61	0.751	0.021	n.s.	2.05 ± 3.79	1.02	0.388	0.025	n.s.
Pen	4.57 ± 4.81	0.79	0.594	0.027	n.s.	4.73 ± 5.09	4.27	<0.01	0.098	Q4 > Q1
Min	207.92 ± 97.78	0.93	0.486	0.031	n.s.	234.56 ± 84.91	0.42	0.738	0.011	n.s.
Min-M	25.09 ± 11.29	0.98	0.446	0.033	n.s.	25.71 ± 8.79	0.19	0.905	0.005	n.s.
	**Women**									
	**Formative Categories (U-18/U-20)**					**Senior Category**				
**Statistics**	**X** **±** **SD**	**Q**			**Differences**	**X** **±** **SD**	**Q**			**Differences**
		**F**	***p***	***η**^**2**^*			**F**	***p***	***η**^**2**^*	
Go	12.29 ± 11.54	0.38	0.912	0.020	n.s.	13.38 ± 12.66	2.14	0.100	0.062	n.s.
PeSh	49.20 ± 24.71	1.97	0.064	0.093	n.s.	53.80 ± 26.22	0.70	0.558	0.021	n.s.
Sa	35.27 ± 20.90	2.69	0.079	0.551	n.s.	47.47 ± 26.90	0.21	0.815	0.040	n.s.
PeSa	30.06 ± 8.51	1.05	0.436	0.324	n.s.	35.40 ± 5.73	0.52	0.610	0.094	n.s.
As	3.35 ± 4.62	0.75	0.633	0.038	n.s.	6.73 ± 7.63	0.91	0.438	0.027	n.s.
To	7.68 ± 7.03	0.57	0.776	0.029	n.s.	7.45 ± 7.96	0.59	0.622	0.018	n.s.
St	1.10 ± 2.57	1.38	0.219	0.067	n.s.	2.32 ± 2.85	4.08	<0.01	0.112	Q2 > Q3
BS	0.34 ± 1.62	0.92	0.495	0.046	n.s.	1.27 ± 2.63	1.40	0.247	0.042	n.s.
Pen	4.36 ± 4.45	1.08	0.381	0.053	n.s.	4.91 ± 4.96	5.67	<0.01	0.149	Q2 > Q3; Q2 > Q4
Min	183.79 ± 94.74	0.55	0.798	0.028	n.s.	204.43 ± 104.64	3.17	<0.05	0.089	Q1 > Q3; Q2 > Q3
Min-M	26.29 ± 12.62	0.79	0.595	0.040	n.s.	26.02 ± 11.30	4.52	<0.01	0.123	Q1 > Q3; Q2 > Q3

“*U-19/U-21,” male youth and junior categories; “U-18/U-20,” female youth and junior categories; “Go,” goals; “PeSh,” percentage of effectiveness in shots; “Sa,” saves; “PeSa,” percentage of effectiveness in saves; “As,” assists; “To,” turnovers; “St,” steals; “BS,” blocked Shots; “Pen,” Penalties; “Min,” minutes played; “Min-M,” minutes played per match; “Cl,” classification; “Q1-Q4/Q8,” birth quartile; “F,” statistic (ANOVA); “p,” level of significance; “η^2^,” eta squared statistic (effect size); “n.s.,” not significant*.

With regard to the relationship between RAE and collective performance (Table 4), an overrepresentation of relatively older players was detected in the semi-finalist teams [χ^2^*(7)* = 17.57, *p* < 0.05] and quarter-finalist teams [χ^2^*(7)* = 25.07, *p* < 0.01] in male formative categories (U-19/U-21) and the quarter-final teams [χ^2^*(7)* = 21.67, *p* < 0.01] in the female formative categories (U-18/U-20). The largest effect size was identified in the female formative categories, and a strong association was found between the variables (*Vc* = 0.67). RAE were found to have no impact in the teams ranked from the 9th to 24th place, both in the male and female formative categories, as well as in the senior teams (*p* > 0.05).

**Table 4:** Quarterly distribution (*n* and %) of birth dates by gender and competitive level according to the final team position.

**Formative categories**						
**Q**	**Men (U-19/U-21)**			**Women (U-18/U-20)**		
	**Semi-finalists**	**Quarter-finalists**	**Bottom sixteen**	**Semi-finalists**	**Quarter-finalists**	**Bottom sixteen**
Q1	15(16.3)	29(24.2)	2(12.5)	2(12.5)	14(29.2)	9(9.5)
Q2	16(17.4)	20(16.7)	4(25.0)	4(25.0)	10(20.8)	10(10.5)
Q3	18(19.6)	20(16.7)	2(12.5)	2(12.5)	5(10.4)	12(12.6)
Q4	10(10.9)	11(9.2)	4(25.0)	4(25.0)	8(16.7)	18(18.9)
Q5	16(17.4)	11(9.2)	3(18.8)	1(6.3)	2(4.2)	8(8.4)
Q6	7(7.6)	10(8.3)	0(0)	1(6.3)	2(4.2)	14(14.7)
Q7	5(5.4)	7(5.8)	0(0)	1(6.3)	4(8.3)	13(13.7)
Q8	5(5.4)	12(10.0)	1(6.3)	1(6.3)	3(6.3)	11(11.6)
χ^2^	17.57	25.07	2.75	6.00	21.67	5.97
*df*	7	7	5	7	7	7
*p*	0.014	0.001	0.738	0.540	0.003	0.543
*Vc*	0.44	0.46	0.41	0.61	0.67	0.25
**Senior category**						
**Q**	**Men**			**Women**		
	**Semi-finalists**	**Quarter-finalists**	**Bottom sixteen**	**Semi-finalists**	**Quarter-finalists**	**Bottom sixteen**
Q1	21(32.8)	17(33.3)	3(18.8)	17(35.4)	0(0)	22(33.8)
Q2	13(20.3)	5(9.8)	5(31.3)	13(27.1)	0(0)	21(32.3)
Q3	9(14.1)	14(27.5)	3(18.8)	8(16.7)	0(0)	10(15.4)
Q4	21(32.8)	15(29.4)	5(31.3)	10(20.8)	0(0)	12(18.5)
χ^2^	6.75	6.65	1.00	3.83	-	6.94
*df*	3	3	3	3	-	3
*p*	0.080	0.084	0.801	0.280	-	0.074
*Vc*	0.32	0.36	0.25	0.28	-	0.33

“*U-19/U-21,” male youth and junior categories; “U-18/U-20,” female youth and junior categories; “Q1-Q4/Q8,” birth quartile; “X^2^,” Chi square; “df,” degrees of freedom; “p,” level of significance; “Vc,” Cramer's V*.

### RAE—Performance Evolution Trends

In Table 5, we show the individual performance trends based on RAE (Q1 and Q4/Q8), by gender and competitive level, at the World Championships between 2005 and 2020. In the formative categories, no general trend over time in individual performance was detected during the 16 seasons (*p* > 0.05), except a downward trend in “goals” in relatively younger male players (14.43 ± 10.11, *y* = −0.82). In the senior categories, the impacts of RAE varied. Relatively older female players performed at a lower level in “assists” (6.41 ± 7.17, *y* = −0.55); while this negative performance trend was still evident in relatively older male players (“percentage of effectiveness in shots” = 56.02 ± 23.97, *y* = −2.20; “percentage of effectiveness in saves” = 33.00 ± 3.67, *y* = −1.63), “assists” (10.76 ± 10.45, *y* = −0.57), and “penalties” (3.68 ± 4.12, *y* = −0.28). Nevertheless, the RAE affected relatively younger men differently than relatively younger women. The data revealed that men born in Q4 demonstrated a worse performance in skills such as “goals” (17.66 ± 10.26, *y* = −1.02), “assists” (9.85 ± 9.64, *y* = −0.50), and “minutes played” (236.98 ± 84.68, *y* = −4.23); while the trend was the opposite in women born at the end of the year (Q4) in “goals” (12.62 ± 11.47, *y* = +1.16); “percentage of effectiveness in shots” (59.14 ± 22.76, *y* = +1.99) and “assists” (7.23 ± 9.02, *y* = +0.93).

**Table 5:** Pairwise comparisons between relatively older (Q1) and relatively younger (Q4/Q8) handball players of the individual performance throughout the last 16 seasons (2005–2020) according to gender and competition level.

	**Men**															
**I.P.S**	**Formative categories (U-19/U-21)**								**Senior category**							
	**Q1**				**Q2**				**Q3**				**Q4**			
	**X ± SD**	***p***	**Slope**	**QI**	**X ± SD**	***p***	**Slope**	**QI**	**X ± SD**	***p***	**Slope**	**QI**	**X ± SD**	***p***	**Slope**	**QI**
Go	17.71 ± 10.87	0.913	0.19	?	14.43 ± 10.11	0.029	−0.82	↓	18.49 ± 11.58	0.253	−0.22	?	17.76 ± 10.26	0.004	−1.02	↓^*^
PeSh	52.06 ± 24.55	0.415	1.15	?	43.86 ± 31.78	0.504	−2.00	?	56.02 ± 23.97	0.009	−2.20	↓^*^	59.24 ± 24.21	0.744	−0.23	?
Sa	20.25 ± 9.81	0.610	−0.10	?	34.67 ± 17.62	0.247	0.93	?	55.80 ± 27.38	0.021	5.02	↑^*^	51.67 ± 35.57	0.856	0.40	?
PeSa	33.00 ± 8.04	0.824	0.71	?	34.33 ± 6.35	0.181	1.97	?	33.00 ± 3.67	0.006	−1.63	↓^*^	36.33 ± 3.06	0.538	0.42	?
As	5.13 ± 5.49	0.727	0.12	?	2.94 ± 4.33	0.118	−0.14	?	10.76 ± 10.45	0.043	−0.57	↓^*^	9.85 ± 9.64	0.286	−0.50	↓^*^
Pen	4.85 ± 5.28	0.114	−0.09	?	5.61 ± 6.34	0.144	0.26	?	3.68 ± 4.12	0.045	−0.28	↔↓	7.17 ± 6.21	0.315	−0.18	?
Min	194.24 ± 83.77	0.187	5.24	?	191.90 ± 90.71	0.425	−4.39	?	233.95 ± 83.44	0.745	−1.12	?	236.98 ± 84.68	0.047	−4.23	↓^*^
Min-M	23.67 ± 10.27	0.686	0.37	?	22.78 ± 10.34	0.189	−0.84	?	25.85 ± 9.10	0.315	0.24	?	26.18 ± 8.82	0.532	−0.07	?
	**Women**															
**I.P.S**	**Formative categories (U-18/U-20)**								**Senior category**							
	**Q1**				**Q2**				**Q3**				**Q4**			
	**X** **±** **SD**	***p***	**Slope**	**QI**	**X** **±** **SD**	***p***	**Slope**	**QI**	**X** **±** **SD**	***p***	**Slope**	**QI**	**X** **±** **SD**	***p***	**Slope**	**QI**
Go	14.12 ± 14.08	0.699	−0.78	?	14.36 ± 13.62	0.934	0	?	14.88 ± 10.23	0.116	0.10	?	12.62 ± 11.47	0.042	1.16	↑^*^
PeSh	40.87 ± 32.23	0.306	−0.15	?	41.50 ± 27.40	0.707	−0.79	?	54.51 ± 26.77	0.794	0.42	?	59.14 ± 22.76	0.049	1.99	↑^*^
Sa	41.56 ± 21.21	0.406	0.31	?	49.67 ± 1.53	0.471	0.21	?	59.43 ± 30.52	0.793	0.64	?	0 ± 0	-	-	-
PeSa	32.20 ± 5.24	0.230	−1.51	?	34.90 ± 1.25	0.477	0.16	?	36.86 ± 5.73	0.741	0.40	?	0 ± 0	-	-	-
As	2.60 ± 2.86	0.951	0.02	?	2.40 ± 2.97	0.882	−0.17	?	6.41 ± 7.17	0.049	−0.55	↓^*^	7.23 ± 9.02	0.036	0.93	↑^*^
Pen	3.84 ± 5.47	0.801	0.06	?	3.53 ± 4.34	0.846	0.02	?	5.05 ± 5.43	0.174	0.01	?	3.95 ± 3.47	0.333	0.14	?
Min	180.99 ± 104.26	0.100	−7.83	?	155.57 ± 82.47	0.901	5.10	?	228.45 ± 105.66	0.673	−1.30	?	164.35 ± 71.03	0.339	1.17	?
Min-M	25.25 ± 13.97	0.103	−0.99	?	20.97 ± 10.93	0.569	0.90	?	28.69 ± 10.78	0.741	0.13	?	21.61 ± 8.11	0.187	0.32	?

“U-19/U-21,” male youth and junior categories; “U-18/U-20,” female youth and junior categories; “Q1,” relative older players; “Q4/Q8,” relative younger players; “p,” significance level; “QI,” qualitative inference; “I.P.S.,” individual performance statistics; “Go,” goals; “PeSh,” percentage of effectiveness in shots; “Sa,” saves; “PeSa,” percentage of effectiveness in saves; “As,” assists; “Pen,” penalties; “Min,” minutes played; “Min-M,” minutes played per match; “?,” very unlikely increase/decrease (unclear change); “↔↑*/↔↓*,” unlikely trivial increase/decrease; “↔↑/↔↓,” trivial increase/decrease; “↑/↓,” substantial increase/decrease; “↑*/↓*,” likely substantial increase/decrease; “-,” No statistical data for this subgroup of the sample.

## Discussion

In the following section, we examine three key findings: (a) the prevalence of RAE and its impacts on performance; (b) the relationship between RAE and evolutionary trends (in 631 Spanish handball players) across the 2005–2020 handball seasons; and, (c) unanticipated finding including the presence of reverse RAE in the “back” players of the male senior category.

### The Prevalence of RAE and Its Relation to Performance

The influence of RAE were observed in all the male player categories, but was only detected in the U-18 and senior categories in the female competitions. This lower incidence of RAE among female players could be explained by the “depth of competition hypothesis” (Baker et al., 2009), which suggests that RAE are likely to be less prevalent because there are fewer female players in competitive handball (Götze and Hoppe, 2021). In Spain, for example, only 36.04% of the 100,368 licences (for handball competitions) were issued for women (Consejo Superior de Deportes. Ministerio de Cultura y Deporte, 2019). Moreover, the official competitions organised by the RFEBM include more teams in categories for men (*n* = 134) than categories for women (*n* = 70). Furthermore, physical and anthropometric factors may influence the performance of male players more because of the physical requirements across playing positions in male handball (e.g., height and weight), which could explain the selection of relatively older players (Camacho-Cardenosa et al., 2018). These same attributes may not be as decisive in affecting players performance in the female categories (Konstantinos et al., 2018).

RAE decreased in the male competitions as the players aged over the seasons between 2005 and 2020, which has been noted in other studies (Wrang et al., 2018; Sá et al., 2020). However, our study revealed a trend was not detected in female categories, which suggests that RAE may vary more in female handball. Indeed, similar results have been reported by Schorer et al. (2009), whilst a reverse impact on RAE have been suggested by others, whereby relatively younger players are overrepresented in the senior categories as reported by Figueiredo et al. (2020). The physical demands throughout the different stages of sport development processes, as well as the level of competition and sociocultural factors, could explain the weak and moderate prevalence of RAE in female team sports, such as handball (Smith et al., 2018).

The influence of RAE on the selection processes for playing positions has been documented (Ibañez et al., 2018), with some researchers suggesting that the impact of RAE may be accentuated in handball because the sport requires a high degree of specialisation (Schorer et al., 2009). In our study, RAE affected most strongly the playing positions of “centre-back” in all male categories, as well as the “wing” in the male formatives and female senior categories. A similarly strong effect on the “wing” position has been also been reported in previous investigations of handball (Karcher et al., 2014; Figueiredo et al., 2020). Wing players are typically shorter, and because they are located in the outer areas of the court (Massuca et al., 2015), they cover the most distance and are the fastest players during match plays (Karcher and Buchheit, 2014). These performance requirements favour relatively older players being selected for this playing position because they require higher levels of physical performance, as demonstrated in tests such as the Yo-Yo Intermittent Recovery Test 1, the vertical jump height Abalakov, or the Barrow speed-agility test (Schwesig et al., 2017; Camacho-Cardenosa et al., 2018). The stronger prevalence of RAE for those playing in “centre-back” positions could be explained, not only by the specific physical characteristics and sport demands required, but also by the favourable psychological characteristics of relatively older players, such as self-confidence and leadership, that could help to enhance players' performances (Chittle et al., 2017). Furthermore, from an environmental perspective, coaches may be more likely to choose older players for these positions (Krahenbühl and Leonardo, 2020).

RAE were found not to have a significant impact on the individual performance parameters of players in the formative categories (U-18/U-19 and U-20/U-21). Although RAE generally affected the selection processes in these stages, the potential for successful performance did not decrease based on the relative age of the athletes (García et al., 2014; Rubajczyk et al., 2017). In international competitions, coaches tend to align players according to technical-tactical criteria (Karcher et al., 2014), depending on the specificity of particular playing positions (Ibañez et al., 2018). In the initial selection process, strict player screening according to specific physical sport criteria (Schorer et al., 2013) could mean that individual performance is not excessively affected by RAE (Bjørndal et al., 2018a). On the other hand, studies have demonstrated a relationship between RAE and individual competition performance in the lower competition categories of handball (de la Rubia et al., 2020a; Krahenbühl and Leonardo, 2020). Nevertheless, the parameter considered was “played time” and, therefore, the impact of RAE on other statistical performance might not have been apparent or appreciated. Interestingly, in the male senior category, relatively younger players (Q4) were found to make more mistakes than those born in Q2 and Q1, whilst in the female senior category, the relatively older players (Q1 and Q2) enjoyed more time on the court than those born in Q3.

Some studies have reported a lower incidence of RAE in the senior category (Cobley et al., 2009; Smith et al., 2018; de la Rubia et al., 2020b). However, in team sports, RAE has been found to also affect competition performance in adult stages (Vaeyens et al., 2005). If coaches focus on immediate results or short-term objectives, they are more likely to favour relatively older players, especially in the formative categories. In Spanish handball, due to its great popularity, this fact could also be explained by secondary environmental factors, such as better training conditions, coaching resources and higher competitive levels (Hancock et al., 2013). This reality fosters the creation of self-strengthening mechanisms in relatively older players who would tend to achieve high performance in a higher proportion than relatively younger players (Barnsley et al., 1992). This is what is known as “The Matthew Effect” (Nolan and Howell, 2010) and highlights how the “when” and “where” could have an impact on performance.

In the analysis of collective competition performance, RAE were found to influence the final team positions in the formative categories of handball (González-Víllora et al., 2015; Arrieta et al., 2016; Rubajczyk et al., 2017). Similar studies have showed a strong correlation between result rankings in youth and senior categories in international competitions (Bjørndal et al., 2018b), which suggests that addressing unequal opportunities in initial player selections is particularly important. From a talent development perspective, this means that during their sporting careers, relatively younger players are able gradually to overcome differences caused by their initial lag in relative age by acquiring technical-tactical, strategic and psychological skills (McCarthy and Collins, 2014). Consequently, by the time they reach the senior category, physical-anthropometric factors become once again equal across the birthdates, and relatively older players no longer have any special advantage over younger players (Bjørndal et al., 2018a). Surprisingly, some studies have reported a reverse RAE, in which relatively younger players are overrepresented in senior categories (Gibbs et al., 2012; Fumarco et al., 2017; de la Rubia et al., 2020b). This, as some have argued, could be explained based on the psychological influence of the additional challenges experienced by the relatively younger players through their developmental journey (McCarthy et al., 2016). For example, Collins et al. (2016) have argued that talented potential can often benefit from, or even need, a variety of challenges to facilitate eventual adult performance, whereby being a later maturer could pose as one such challenge. However, as with the other proposed explanations for the impacts of the RAE in sport, they claim should be treated with caution until it has been substantiated by more empirical evidence.

### The Relationship Between RAE and Evolutionary Trends

Our results are congruent with other studies of the impact of the RAE in national talent development programmes in handball in two key ways: (1) RAE appeared to decrease progressively throughout the developmental stages (see also Bjørndal et al., 2018a; Figueiredo et al., 2020; Sá et al., 2020); and, (2) the presence of relatively younger players was found to increase as the age of the players increased over the seasons (see also Wrang et al., 2018). However, as noted earlier, our analysis extended beyond the approaches that have been used in previous research because it also investigated the potential impacts of the RAE across *long-term* player performance. Specifically, our study revealed no performance trends affected by the RAE in the formative categories throughout the 16 seasons analysed, except a downward trend observed in “goals” in relatively young male players, while in the senior category the trends are different by gender: in men, a worsening of performance was identified in both the relatively older and the younger players; and, in women, the trend did not vary for relatively older women, but a better performance was observed in relatively younger women.

In men's competitions, the RAE was not the main factor affecting long-term individual performance. The lack of the RAE in male players within the RFEBM TID system (Gómez-López et al., 2017; Camacho-Cardenosa et al., 2018) seems to indicate that the selection processes are organised around criteria that are far from the physical and maturational component. Therefore, a recruitment methodology based on game-specific factors and technical-tactical skills could mean that all players start with the same opportunities to compete in specialised international contexts (Karcher et al., 2014). Furthermore, a training of coaches oriented to the promotion of these strategies, as advocated Krahenbühl and Leonardo (2020), would move the selection processes away from the immediate and current performance of the player (Bailey and Collins, 2013), favouring competition performance not biassed by the RAE. Indeed, Bjørndal et al. (2018a) showed that, although international team selections favour relatively older players, they do not strongly affect the possibility of achieving a long-term individual performance internationally.

At the higher competitive levels (i.e., senior category), those female players who achieved the best performance were relatively younger. Therefore, RAE did not appear to limit the selections or performance of female players over time. Among other factors (e.g., depth of competition and reduced pool of players), this trend could be explained by the relatively earlier start of pubertal maturation compared to males. The fastest growth rate in females occurs between the ages of 11 and 14 years (Malina et al., 2015), and the maturity status tends to equalise earlier than males. This means that there are likely to be almost no differences in the physical maturity of the female players at higher competitive levels (Smith et al., 2018), as well as that relatively younger female handball players could potentially achieve better sport performance earlier than their male counterparts. This positive evolution in relatively younger female players would highlight the “underdog effect” with regard to competition performance (Fumarco et al., 2017). The female players born at the end of the year would overcome physical and maturational differences and benefit, over time, from an acquisition of technical-tactical and psychological skills superior to those acquired by relatively older players (McCarthy et al., 2016).

### Unanticipated Finding

Surprisingly, reverse RAE were detected among players in the “back” position in the male handball senior category, whereby relatively younger players were favoured over relatively older counterparts. This position requires strong and tall players (Karcher and Buchheit, 2014) and is even affected by the concept of “handedness,” especially on the right side of the court (Schorer et al., 2009). However, a possible explanation could be based on environmental factors. The relatively younger players in this instance may have benefited from favourable training conditions in the specific context of the Spanish handball game setting—a model which focuses strongly on individual techniques and tactical training rather than physical qualities alone (Román Seco, 2008). The development of technical-tactical skills may have helped to alleviate differences between them and their more mature peers. Therefore, the placement in this position of players with superior visual perception skills, linked to the offensive collective game standards of Spanish handball, may result in a lower component of specialisation players in the “back” position (Román Seco, 2009). Further, if the versatility of this kind of player is considered, it could result in relatively younger players being selected for the “back” position, unlike for other positions. Future research should aim to analyse whether those players who play the “back” position in the senior category are, perhaps, late maturers who require a longer period to develop physically.

### Limitations

First, a relatively small sample size, in terms of the birthdate distribution (Q), has meant that some statistical analyses could not be carried out due to a low frequency (<5), especially with the stratification of the sample by gender, category and playing position. Second, the individual performances of players could not be accurately compared due to the lack of a general performance indicator, such as a “performance index rating—PIR” (Rubajczyk et al., 2017; Ibañez et al., 2018) that would have allowed us to analyse the impact of RAE more objectively. Third, the lack of individual performance data from other international competitions (European Handball Championships or Olympic Games) meant it was not possible to examine or compare the relationship between RAE and performance in, or across, other contexts. Fourth, the study considered only the internal context of national teams and the associated performance of the players involved. This meant that we were not able to compare other prior selection processes in other environments (such as clubs, regional teams, and follow-up talent concentrations). Fifth, some of the Spanish national teams did not compete in the World Handball Championships (i.e., 2005–2006: male U-19 and female U-18; 2011–2012: female U-18; and, 2013–2014: female U-18 and female U-20). The 2019–2020 cycle was also interrupted by the suspension of official handball competitions due to by COVID-19, thus the female U-18 and female U-20 teams did not compete.

## Conclusions

The present study was designed to address the absence of longitudinal studies by exploring the relationship between RAE and competition performance, as well as providing an overview of how the relationship has evolved over time. This is an especially important focus in team sports, such as handball, where performance is multifactorial and difficult to assess. Our findings showed that RAE were a determining factor in the player selection processes conducted by the RFEBM Spanish handball teams for the World Handball Championships held between 2005 and 2020. Although some variability of the prevalence of RAE were observed by gender, competitive level, and playing position, RAE proved to be a critical factor shaping the processes of talent identification and development programmes in this elite professional sport organisation.

However, it was not clear from the data if RAE had an impact on the individual and collective performance of players according to the variables analysed. In the formative categories, a high percentage of relatively older players appeared to favour better ranking success. In the senior category, RAE affected those born in the first months of the year only in terms of a higher number of minutes played. Differences in individual performance between the relatively younger Spanish handball players and the relatively older players gradually lessened through the eight biannual cycles we analysed, except in the female competitive categories. Greater variability in the impact of RAE, as well as sociocultural and sport-specific factors associated with handball competitions, could be responsible for our failure to identify a clear RAE-linked long-term performance trend in Spanish female handball.

## Data Availability Statement

The original contributions presented in the study are included in the article/supplementary material, further inquiries can be directed to the corresponding author.

## Author Contributions

All authors listed have made a substantial, direct and intellectual contribution to the work, and approved it for publication.

## Conflict of Interest

The authors declare that the research was conducted in the absence of any commercial or financial relationships that could be construed as a potential conflict of interest.
